# The Sufferings of Christ

**Published:** 1887-10-08

**Authors:** 


### WORDS OF CONSOLATION.

[Communications for this column should be addressed, "Chaplain," care of the Editor of THE HOSPITAL, who will gratefully welcome any suggestions or inquiries from matrons, nurses, and the staff generally.]

### LIII.—THE SUFFERINGS OF CHRIST.

*(For Reading to the Sick.)*

FROM the Agony and Bloody Sweat, from the lonely Garden and the burden of Gethsemane, we must pass on to another branch of Our Lord's sufferings for us. We must pass through the remainder of that night with Him, and on to the dawn of the first Good Friday. The latter part of the night was very different from the beginning. Though He suffered throughout, the suffering was of a different kind from what He experienced in the Garden. Perhaps the words which best comprehend His treatment and conduct from the time of the Agony to the Crucifixion are given in Isaiah liii, 7: "He was oppressed and He was afflicted, yet He opened not His mouth; He is brought as a lamb to the slaughter, and as a sheep before his shearers is dumb, so He openeth not His mouth." (The fifty-third chapter of Isaiah should be read most carefully if you would know the sufferings as a Christian should know them.)

All through the long hours, while Our Lord was before Annas and Caiaphas, and in the judgment-hall, this one feature in His conduct is especially noted, namely, the meekness and silence of the Lamb of God, bearing all things for us without a murmur. When He was reviled reviling not again; oppressed and afflicted by the very creatures He was bleeding to save, yet not a word of reproach escaped His lips, not a single effort did He make to resist them, because when He rose from His knees for the third time in the Garden, it was with a will completely set to endure all that could be heaped upon Him without complaining. Jesus had really to pass through four different trials before He was crucified. One before Annas, another before Caiaphas, then before the whole Council and the chief priests, then before Pilate. Besides these He was taken before King Herod. The accounts should be read separately in order to discern the very kindness and love there was in the periods of silence, and the meekness, yet the majesty of His answers, when He felt constrained to speak. While He answered nothing when assailed with every kind of false accusation which envy, hatred, and malice could hurl against His own innocent soul, one instance may suffice to prove that He spoke only to vindicate His Father's Name and honour; only when put upon His oath did He once more declare the fact of His Godhead, and the judgment committed to Him as the Son of Man (see MATT. xxvi, 62—64). Then followed a storm of rage. "We have heard His blasphemy, He is guilty of death; and they spit upon Him, and buffeted him, and smote Him with the palms of their hands." What a contrast! Behold the meekness of the Lamb, and the increasing rage of His persecutors.

Do you know what it is ever to be provoked by meekness? When you have been angry with people or tried to irritate them, and they would not let you put them out of temper? Ah! just that determination on their side not to be provoked may have increased your own irritation. You felt that they were the true possessors of a power you had lost, and you envied them, and were very sorry afterwards for having sinned yet more in the presence of so much meekness. This may help you to understand the increasing violence under which Christ's sufferings were aggravated. At the same time let us learn, whenever we feel annoyed in the presence of those who are examples of meekness and self-control, never to degrade ourselves by manifesting impatience. The ornament of a meek and quiet spirit is, in the sight of God, of great price. There are times when silence is the best and the kindest answer possible.

We desire to recommend "Our Work for Christ," a little book for hospital nurses, by M. A. Morrell, published by Rivingtons.

